# Pre‐Incision Time Intervals and Outcomes of Free Flap re‐Exploration: A Retrospective Single‐Center Study

**DOI:** 10.1002/micr.70292

**Published:** 2026-09-11

**Authors:** Alexander Fiedler, Nesrin Ibrahim, Maria Füth, Tatjana Katharina Fricke, Sonja Verena Schmidt, Felix Reinkemeier, Flemming Puscz, Yonca Steubing, Marcus Lehnhardt, Christoph Wallner, Marius Drysch

**Affiliations:** ^1^ Department of Plastic Surgery Ruhr University Bochum, BG University Hospital Bergmannsheil Bochum Germany

**Keywords:** free flap, microsurgery, operating room efficiency, perioperative delay, re‐exploration, salvage, thrombosis

## Abstract

**Background:**

Timely re‐exploration is critical when free flaps show vascular compromise. However, the relative contribution of modifiable pre‐incision delays after surgery registration and opportunities to shorten time to re‐exploration within the recognition‐to‐incision pathway after flap ischaemia recognition remain uncertain. This study aimed to evaluate whether pre‐incision delays are associated with free‐flap salvage failure and to identify potential workflow targets for quality improvement.

**Methods:**

We conducted a retrospective, single‐center analysis of 113 free‐flap revision surgeries performed over the past 6 years. Time from registration to incision was partitioned into arrival latency (ward to operating room (OR)), anesthesia lead time, operating‐room preparation, and surgery lead time. Operative duration was assessed separately. The primary outcome was salvage versus failure (total loss).

**Results:**

Total flap loss occurred in 14.2% of re‐explorations. Median registration‐to‐incision time was similar between salvage and failure (78 min vs. 77 min, ±30 min, *p* = 0.89). Arrival latency (19 min vs. 20 min, ±17 min, *p* = 0.89), operating‐room preparation (25 min vs. 22 min, ±12 min, *p* = 0.66) and door to incision (52 min vs. 53 min, ±23.5 min, *p* = 0.5) showed no difference. Failures trended toward longer anesthesia lead time (28 vs. 38 min, ±16.7 min, *p* = 0.48) and operative duration (83 vs. 109 min, ±75 min, *p* = 0.12). Night‐time operations were not associated with failure, but weekend cases showed a higher proportion of failure (25% vs. 12%, *p* = 0.06, trend only).

**Conclusions:**

In this cohort, pre‐incision delays (arrival and OR preparation (prep)) were not associated with failure. Signals instead pointed toward patient and anesthetic complexity and technically challenging salvage procedures. Thus, quality improvement attempts should prioritize rapid recognition/decision pathways and anesthesia‐surgery coordination rather than focusing solely on logistics between ward and incision.

## 1 Introduction

Free‐flap reconstruction has become a standard technique in reconstructive microsurgery, with overall success rates exceeding 95% (Shen et al. 2021; Haughey et al. 2001; Disa et al. 2001). Unlike skin grafts, which depend on diffusion and neovascularization from the wound bed, free flaps maintain their own arterial and venous supply, microsurgically anastomosed to recipient vessels (Min et al. 2022). Despite standardized protocols and experienced teams, between 5% and 14% of free flaps require re‐exploration (Shen et al. 2021; Haughey et al. 2001; Disa et al. 2001) and 2%–5% ultimately fail (Shen et al. 2021; Liu et al. 2019; Bigdeli et al. 2019).

To reduce complications of ischaemia reperfusion injuries as well as vasospasms in free flaps, different approaches such as surgical delay (Cinpolat et al. 2014), ischemic preconditioning (Lee et al. 2022) and pharmacological adjuncts (Soares et al. 2019) have been tested. These approaches can reduce risk in selected contexts, but when mechanical failure occurs, most commonly because of thrombosis, kinking, or torsion of the pedicle, timely surgical re‐exploration is the only definitive option to prevent flap loss (Travis et al. 2023; Bui et al. 2007).

Because ischaemia‐reperfusion injury, vasospasm, and thrombus propagation are time‐dependent processes, the principle “time is tissue” has shaped decades of microsurgical practice (Rogoń et al. 2024; Williams et al. 2004). Accordingly, postoperative care emphasizes early detection and rapid escalation. Many programs adopt intensive monitoring for 1–5 days postoperatively, with flap checks performed hourly to every 4 h, depending on institutional protocol and patient status (Cornejo et al. 2013; Kampshoff et al. 2025). Additionally, structured nurse‐led observations complement physician assessments in several models (Cornejo et al. 2013). Further, early recognition and a low threshold for return to the operating room are warranted. When uncertainty exists, prompt re‐exploration over prolonged observation is prioritized (Novakovic et al. 2009). Reported salvage rates decline from approximately 62%–21% when compromise is first recognized after 16 h, reinforcing this approach (Yang et al. 2007). Consistent with these principles, most arterial and venous failures occur within the first 48 h after the initial operation (Novakovic et al. 2009; Zoccali et al. 2017).

However, while prior studies confirm that long total delays are detrimental, the relative contribution of individual in‐hospital time segments after recognition remains unclear. Once the decision to return to the operating room is made, the subsequent chain from activation and transport to anesthesia induction and surgical set‐up is often treated as a single block of time. It remains uncertain which portion of this pre‐incision interval truly drives outcomes and which steps offer realistic opportunities for improvement (Shen et al. 2021).

We therefore reassessed the time‐is‐tissue paradigm, evaluating whether time affects outcomes and identifying which discrete minutes within the pre‐incision pathway carry the greatest impact. Our objective was to quantify each segment of the registration‐to‐incision delay and to identify where meaningful variance and thus potential improvement actually resides. We hypothesized that the logistical interval acts as a cumulative “silent delay” that influences flap survival despite remaining clinically non‐apparent. By analyzing each step from activation to incision in a retrospective, single‐center cohort, we sought to generate benchmark medians to guide targeted quality‐improvement strategies that support swift re‐exploration.

## 2 Methods

### 2.1 Study Design and Population

We conducted a retrospective, single‐center study including all adult patients who underwent operative re‐exploration for suspected vascular compromise of a free flap at our institution between January 2020 and June 2025. Planned procedures such as debridement of already failed or non‐viable flaps were excluded. All 113 cases included represented genuine unplanned re‐explorations with active salvage intent.

### 2.2 Postoperative Monitoring Protocol

All patients undergoing free‐flap reconstruction received standardized postoperative flap monitoring performed by physicians. Clinical assessments, including skin color, temperature, capillary refill, and handheld Doppler signal, when necessary, were conducted hourly for the first five postoperative days, without reduction in frequency between day‐ and night‐shifts. From postoperative day six onward, structured monitoring was formally concluded and patient mobilization initiated, in accordance with institutional protocol. This standardized, physician‐led surveillance protocol was applied uniformly across the entire study period and formed the basis for clinical recognition of vascular compromise triggering re‐exploration.

### 2.3 Definition and Outcomes

Flap salvage was defined as a viable flap or only small partial loss at discharge. Whereas failure was defined as total flap loss resulting in the need for amputation, another flap, or overall subsequent reconstructive surgery.

We defined the hypothesized silent delay as the interval spanning earliest emergency registration to incision. The arrival latency was the measured time from emergency registration to patient arrival in the operating room area. Further, OR Prep Time was defined as time for surgical setup and instrument preparation. The door‐to‐incision time is a surrogate marker encompassing the total duration from OR arrival to surgical start. Additionally, Anesthesia Time was defined as the interval from patient arrival to “system readiness,” where “system readiness” was the later of anesthesia clearance or the OR column start time, accounting for parallel processes. The surgery lead time was measured from complete system readiness to incision, reflecting final coordination efficiency.

We extracted time stamps from the electronic patient record (EPR). Implausible or negative intervals were verified against OR source logs and, when discrepancies could not be reconciled, excluded from analysis.

### 2.4 Statistical Analysis

All statistical analyses were conducted using Python 3.9.7 with Pandas 1.4.2 for data manipulation, NumPy 1.22.3 for numerical computing, SciPy 1.8.0 for statistical tests, and Matplotlib 3.5.1 and Seaborn 0.11.2 for visualization.

#### 2.4.1 Descriptive Statistics

Continuous variables are reported as median [IQR] given right‐skewed distributions; means were additionally computed for robustness. Categorical variables are reported as counts and percentages. Given non‐normality, Mann–Whitney *U* tests (two‐sided, *α* = 0.05) compared Salvage vs. Failure for each core interval (Silent Delay, Arrival Latency, OR Prep, Door‐To‐Incision, Anesthesia Lead Time, Ready‐To‐Incision, Surgical Duration). For proportions across categories (time of day, weekday/weekend, OR workload, ward), we used chi‐square tests of independence. For quartile analyses (Silent Delay and Surgical Duration), quartiles were constructed and overall chi‐square tests were complemented by exploratory pairwise 2 × 2 chi‐square tests between categories (e.g., Q1 vs. Q4). Fisher's exact test was used in place of chi‐square for 2 × 2 tables with expected cell counts lower than 5.

#### 2.4.2 Temporal and Systems Variables

Time of day was defined as Regular Hours (07:00–15:59) or After Hours (16:00–06:59) based on time of patient registration; Weekday status as Monday–Friday vs. weekend. OR workload was derived from the number of parallel rooms as Low (< 2 ORs) vs. High (≥ 2 ORs). Ward of origin was consolidated into Plastic Surgery ICU, Plastic Surgery Ward, and Other Wards.

#### 2.4.3 Correlation Analysis

For an overview heatmap, categorical variables were one‐hot encoded; Pearson correlations were computed on the combined matrix of continuous measures and dummies to visualize associations among process and system variables. This is descriptive and hypothesis‐generating.

## 3 Results

### 3.1 Cohort Characteristics

Over the study period, 113 flaps out of 470 returned to the operating room for suspected free‐flap compromise and met inclusion criteria. The indication for surgical revision was based on clinical suspicion of flap compromise. Cases in which exploration revealed hematoma or infection in the absence of vascular compromise were not included in the study cohort. Overall, 14.2% of salvage attempts and thus 3.4% of flap reconstructions performed resulted in total flap loss. Patient demographics, flap types, and indications for re‐exploration were broadly comparable between salvaged and failed flaps. The most frequently utilized flap was the anterolateral thigh (ALT) flap (42.5%), followed by the latissimus dorsi flap (21.2%), while abdominal‐based flaps, including deep inferior epigastric perforator (DIEP) and muscle‐sparing transverse rectus abdominis myocutaneous (MS‐TRAM) flaps, accounted for a smaller proportion of reconstructions (Table 1). Notably, our cohort composition was skewed toward extremity reconstruction rather than breast reconstruction (Table 2). Extremity reconstructions carry higher re‐exploration and complication rates than flaps used for breast reconstruction (Arakelyan et al. 2022; Vemula et al. 2017) which likely explains the comparatively high revision volume observed in our cohort.

**TABLE 1:** Distribution of free flap types used for microvascular reconstruction.

Flap type	*n* (%)
ALT	48 (42.5)
Latissimus dorsi	24 (21.2)
DIEP (single)	11 (9.7)
MS‐TRAM	6 (5.3)
DIEP (double)	5 (4.4)
SCIP	5 (4.4)
TFL	4 (3.5)
Gracilis	3 (2.7)
Other	7 (6.2)

*Note:* Values are presented as absolute numbers with corresponding percentages of the total study cohort (*n* = 113).

**TABLE 2:** Distribution of recipient sites of free flaps.

Recipient site	*n* (%)
Lower extremity	65 (57.5)
Breast	20 (17.7)
Upper extremity	18 (15.9)
Trunk (non‐breast)	8 (7.1)
Head/neck	2 (1.8)

*Note:* Values are presented as absolute numbers with corresponding percentages of the total study cohort (*n* = 113).

Further an analysis between muscle and perforator flaps revealed no difference in flap loss. Among muscle flaps, 28 were salvaged, no partial losses occurred, and 4 resulted in total loss (total loss rate 12.5%; any loss 12.5%). Among perforator flaps, 67 were salvaged, 2 partial losses occurred, and 12 resulted in total loss (total loss rate 14.8%; any loss 17.3%). Fisher's exact test showed no statistically significant difference for either any loss (OR = 0.68, *p* = 0.776) or total loss alone (*p* = 1.0; Table S1).

### 3.2 Distribution of Preoperative Delays

**FIGURE 1:**
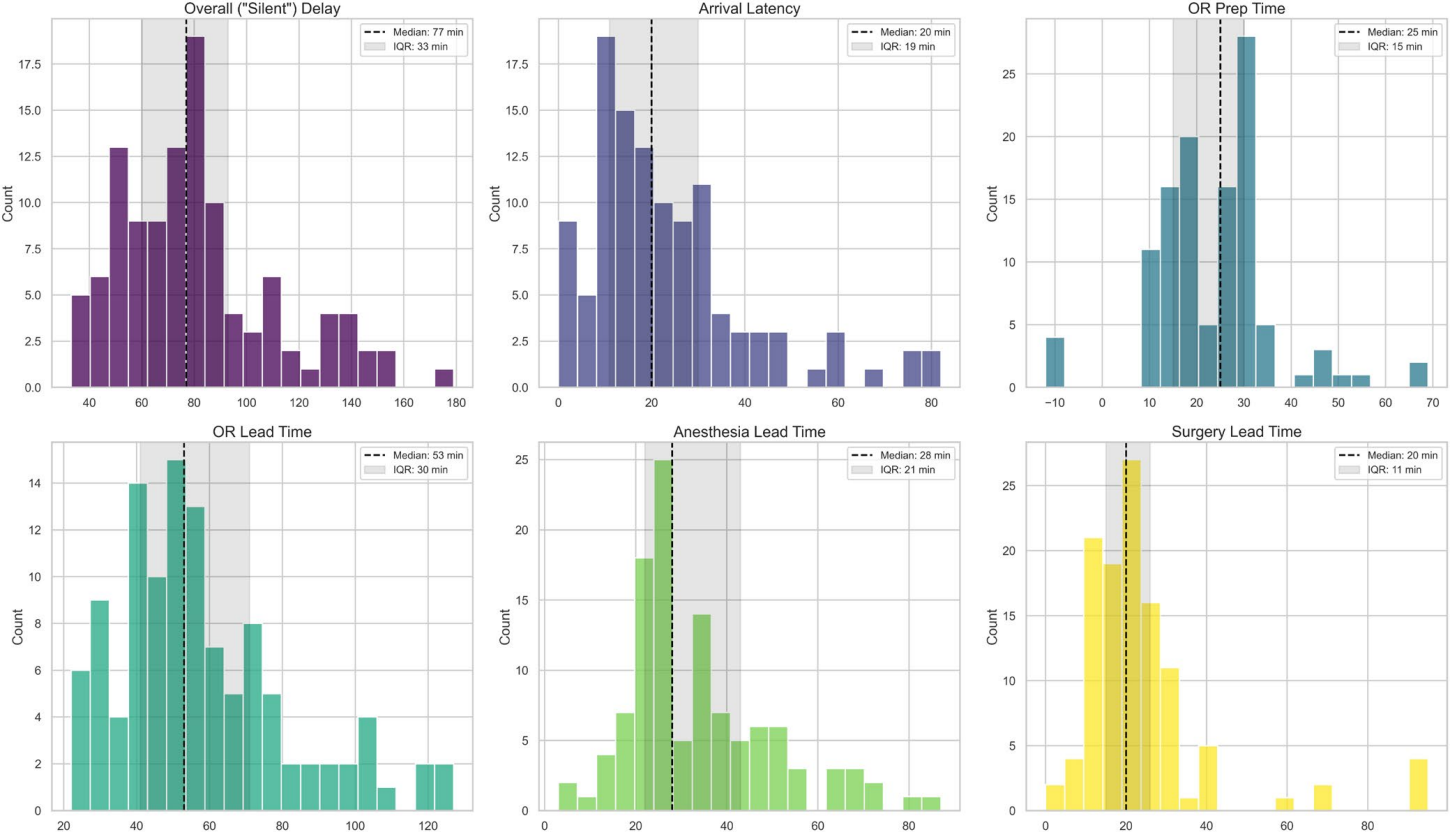
Distributions of pre‐incision (“silent”) delay and its components in free‐flap re‐exploration. Panels show histograms (counts) for overall (“silent”) delay (registration‐to‐incision), arrival latency, OR preparation time, OR lead time (ready to incision), anesthesia lead time, and surgery lead time (minutes). The vertical dashed line marks the median and the gray band denotes the interquartile range (IQR) for each metric.

In case of re‐exploration, the overall registration‐to‐incision, here termed “silent delay,” was 77 min (IQR 33 min; ±30 min; Figure 1). Within this interval, arrival latency, the interval from registration until the patient reached the operating room site, was 20 min (IQR 19 min; ±17 min). Following arrival, operating‐room preparation time, covering surgical set‐up including instruments and microscope, was 25 min (IQR 15 min, ±12 min). Subsequently, OR lead time (door‐to‐incision), the period from arrival in the operating room until incision that includes anesthesia optimization and final surgical checks, was 53 min (IQR 30 min; ±23.5). In parallel, anesthesia lead time, measured from operating‐room arrival until anesthesia declared readiness using the later of anesthesia clearance or OR system readiness to reflect parallel processing, was 28 min (IQR 21 min; ±16.7 min). Finally, surgery lead time, the interval from full system readiness to incision reflecting final team coordination, was 20 min (IQR 11 min; ±17 min).

### 3.3 Global Timelines and Partitioned “Silent Delay”

From the decision to re‐explore to the subsequent incision, the composite pre‐incision interval was divided into distinct process phases, including arrival latency, anesthesia preparation, operating‐room preparation, and surgical set‐up. Across the cohort, distributions of arrival latency, anesthesia preparation, and operating‐room preparation were broadly comparable between salvaged and failed flaps, while operative duration and anesthesia lead time tended to be longer among failures (Table 3).

**TABLE 3:** Process intervals and surgical duration by salvage outcome.

	Salvage median	Salvage IQR	Failure median	Failure IQR	Overall median	Overall IQR
Silent delay	78	37	77	23	77	33
Arrival latency	19	20	20	10	20	19
OR prep time	25	14	22	15	25	15
OR lead time	52	30	55	17	53	30
Anesthesia lead time	28	20	38	22	28	21
Surgery lead	20	11	25	9	20	11
Surgical duration	83	69	109	11	83	72

*Note:* Median times and IQRs (minutes) for pre‐incision “silent delay” components and total surgical duration in salvaged versus failed free flaps and in the overall cohort.

**FIGURE 2:**
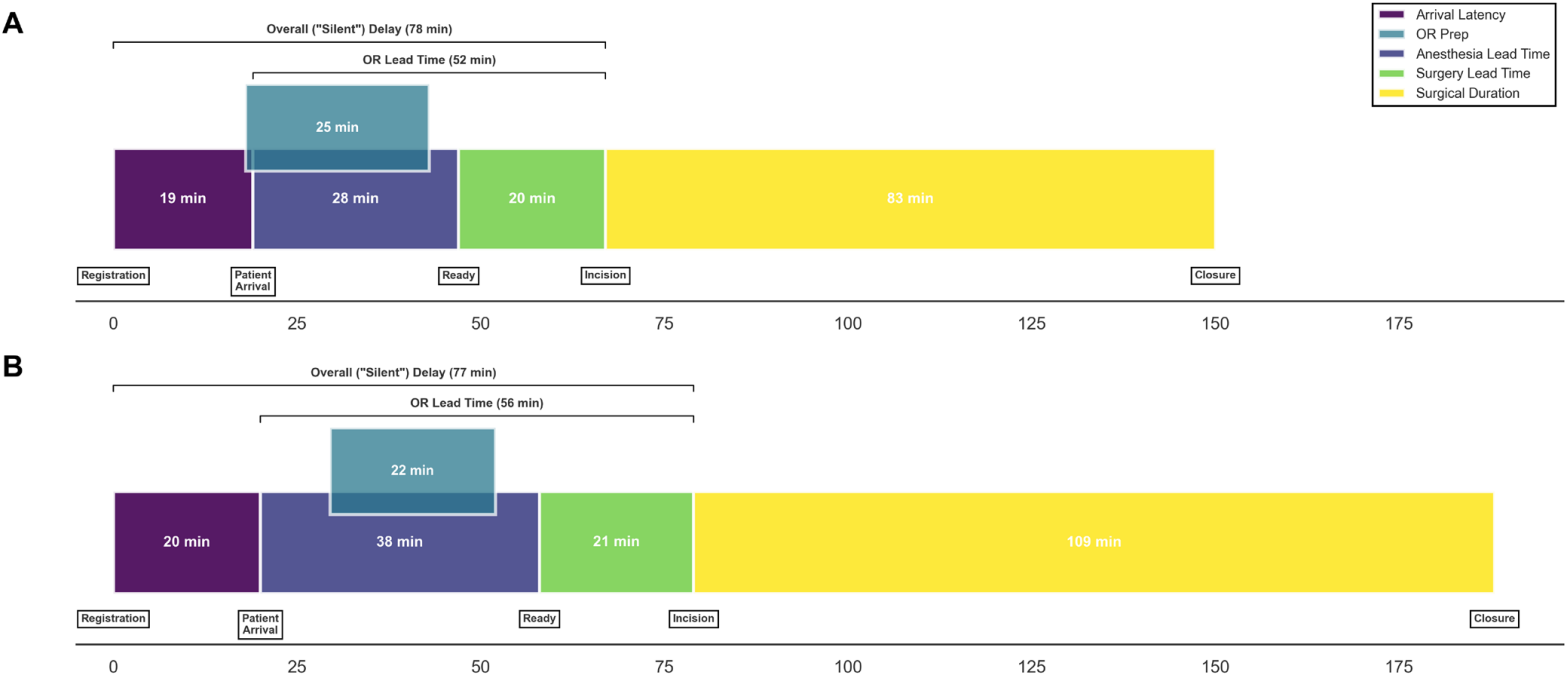
Proportional “silent delay” by outcome among patients undergoing free‐flap re‐exploration. (A) Bar illustrates cases with flap salvage, and (B) illustrates cases that resulted in flap loss. Each horizontal bar is drawn to scale and segmented to show the median duration in minutes of the pre‐incision intervals that contribute to the silent delay. The overall (“silent”) delay spans the sum of different times from arrival to incision (A: 78 min; B: 77 min).

**FIGURE 3:**
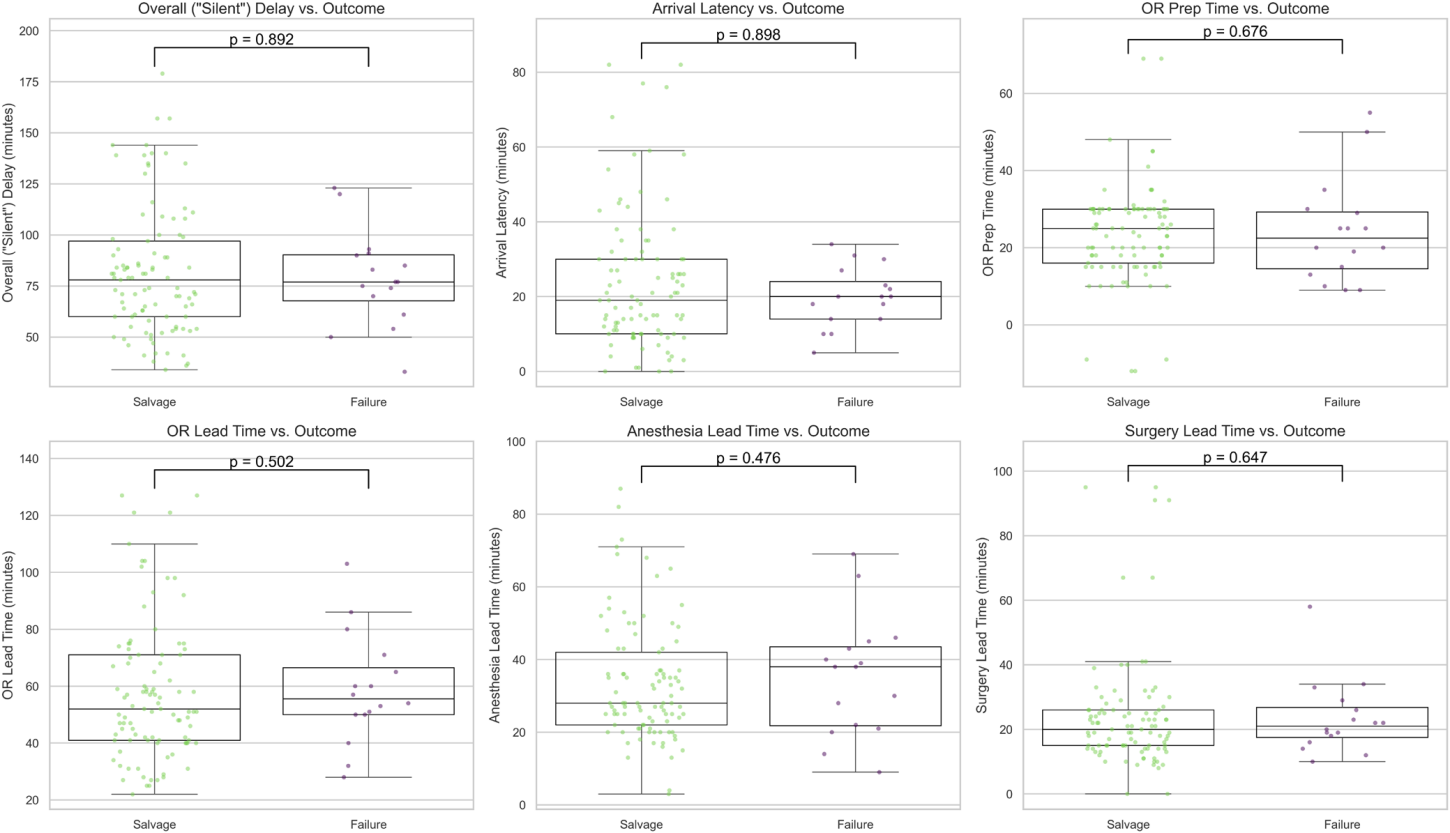
Registration‐to‐incision (“silent delay”) and component intervals by flap outcome in free‐flap re‐exploration. The six panels compare salvage vs. failure using box‐and‐whisker plots: (top left) overall (“silent”) delay, (top middle) arrival latency, (top right) OR preparation time, (bottom left) OR lead time (Ready to Incision), (bottom middle) anesthesia lead time, and (bottom right) surgery lead time. Boxes show the median (center line) and interquartile range (IQR). Whiskers are drawn to Q1 and Q3; points show individual observations (minutes). Group comparisons are annotated with *p*‐values; no differences reached statistical significance (all *p* > 0.05).

Because previous studies have linked total delay to reduced salvage rates, the time from registration to incision was specifically examined (Figure 2). Median values were 78 min for salvaged cases and 77 min for failures, showing no significant difference in central tendency (Figure 3).

To investigate where time is spent during the preoperative process and whether specific intervals within the process significantly influence flap outcomes, we compared individual pre‐incision intervals between salvage and failure cases. Arrival latencies were broadly comparable, with no clear separation between groups providing no evidence of a meaningful difference (Figure 3). After the arrival of the patient, different processes, like anesthesia preparation, take place. These processes could influence flap physiology through systemic factors (e.g., hypothermia, hemodynamic instability) or procedural factors (e.g., challenging induction, positioning delays). For that reason, we analyzed the time from door‐to‐incision (OR Lead Time) including anesthesia and surgery lead time, and if any delay within that time could worsen patients' or flaps' outcome. This interval was similar in failures and salvages (52 min vs. 55 min, ±23.5, *p* = 0.5).

To explore whether a specific component within this window contributed more strongly to outcome variation, anesthesia lead time was analyzed separately. Anesthesia lead time was longer in failures than in salvages (28 min vs. 38 min, Δ = 10 min, ±12 min, *p* = 0.476). As anesthesia lead time and surgery lead time combined are the OR lead time, we further analyzed the Surgery Lead time, which was similar (20 min vs. 25 min, ±17 min, *p* = 0.647).

Additionally, operating technical assistant (OTA) preparation time, used as a surrogate for team experience and operational workload, showed minimal variation between groups (27 min vs. 26 min, ±12 min, *p* = 0.68).

Finally, we evaluated whether longer operative duration was associated with a higher risk of flap failure. Median surgical time was 109 min in failed cases compared with 83 min in salvaged cases (Δ = 26 min, *p* = 0.129). When surgical duration was stratified by quartiles, a *U*‐shaped association with outcome emerged: both the shortest and the longest procedures exhibited higher failure rates.

### 3.4 Temporal Patterns: Hour‐Of‐Day and Day‐Of‐Week

As no specific interval within the in‐hospital process showed a significant effect on flap outcome, we next examined potential temporal influences, including the timing of re‐exploration during the day and across the week. Re‐explorations occurred throughout the 24‐h cycle, with the highest case frequency observed around 7:00.

**FIGURE 4:**
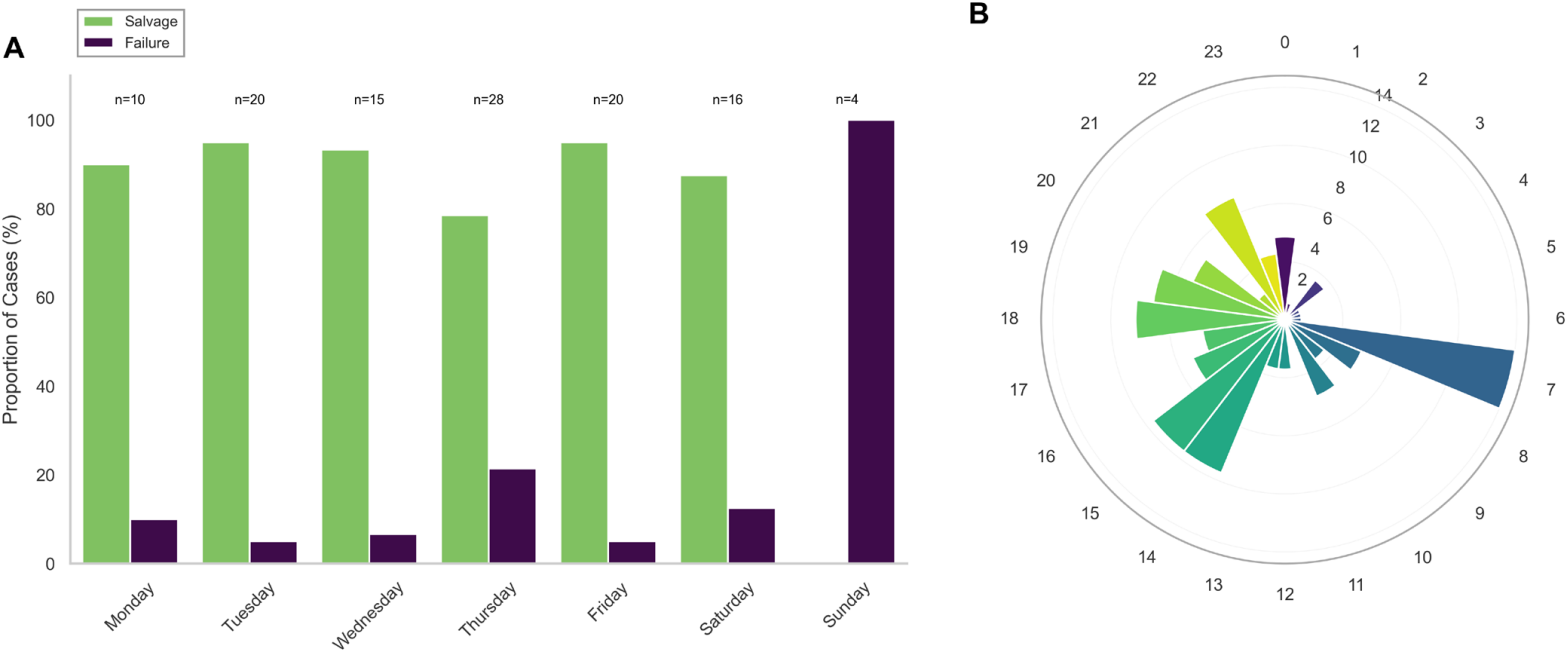
Timing of free‐flap re‐explorations and outcomes. (A) Proportion of salvage (green) and failure (purple) by day of week. Values above bars indicate the number of re‐explorations performed on that day (n). Bars show within‐day outcome proportions (i.e., percentages sum to 100% per day). Note the small Sunday sample (*n* = 4), for which all cases failed. This should be interpreted with caution. (B) Radial histogram of re‐exploration start times by clock hour (0–23). Wedge angle is centered on each hour. The radius encodes frequency (greater radius = more cases). Events are concentrated in daytime hours with comparatively few overnight cases. All counts are cases. Percentages are within‐day proportions.

Night‐time operations (22:00–06:00) were not associated with increased failure rates. However, weekend procedures demonstrated a higher proportion of failures compared with weekday operations (25% vs. 12%; *p* = 0.06). All four salvage procedures performed on Sundays resulted in flap loss (Figure 4), although pre‐incision logistics on weekends were comparable to weekdays (median 68 vs. 77 min; Mann–Whitney *U*, *p* = 0.310), and off‐hours case frequency did not differ significantly (Fisher exact, *p* = 0.179; Table S2). As shown in Table 4, all Sunday re‐explorations occurred ≥ 48 h after index flap surgery, with two cases between 48 and 72 h and two cases beyond 72 h.

**TABLE 4:** Distribution of cases according to weekday of occurrence and time interval from onset (0–24, 24–48, 48–72, > 72 h).

Weekday	0–24 h	24–48 h	48–72 h	> 72 h	Total *n* (%)
Monday	8	0	0	2	10 (8.8)
Tuesday	5	12	0	3	20 (17.7)
Wednesday	7	4	3	1	15 (13.3)
Thursday	16	9	1	2	28 (24.8)
Friday	9	8	0	3	20 (17.7)
Saturday	1	10	1	4	16 (14.2)
Sunday	0	0	2	2	4 (3.5)
Total *n* (%)	46 (40.7)	43 (38.1)	7 (6.2)	17 (15.0)	113 (100)

*Note:* Values represent the number of cases observed within each time interval from primary flap surgery.

### 3.5 Workload, Location of Care, and Ward Effects

Temporal patterns tell only part of the story. Workload intensity and available expertise also affect outcomes. In this cohort, operating room workload at the time of re‐exploration showed no association with failure. Low workload (< 2 ORs running) had 7 failures within 50 salvage operations (14.0%) and high workload (≥ 2 ORs) had 9 out of 63 salvage operations fail (14.3%, *p* = 1.00). Likewise, the patient's location at the time of flap compromise was not associated with different failure proportions as similar salvage rates were assessed across other wards 3/24 (12.5%), the Plastic Surgery ICU 4/23 (17.4%), and Plastic Surgery ward 9/66 (13.6%) (*p* = 0.875). Failure rates were comparable across ward locations and operating‐room workload categories.

**FIGURE 5:**
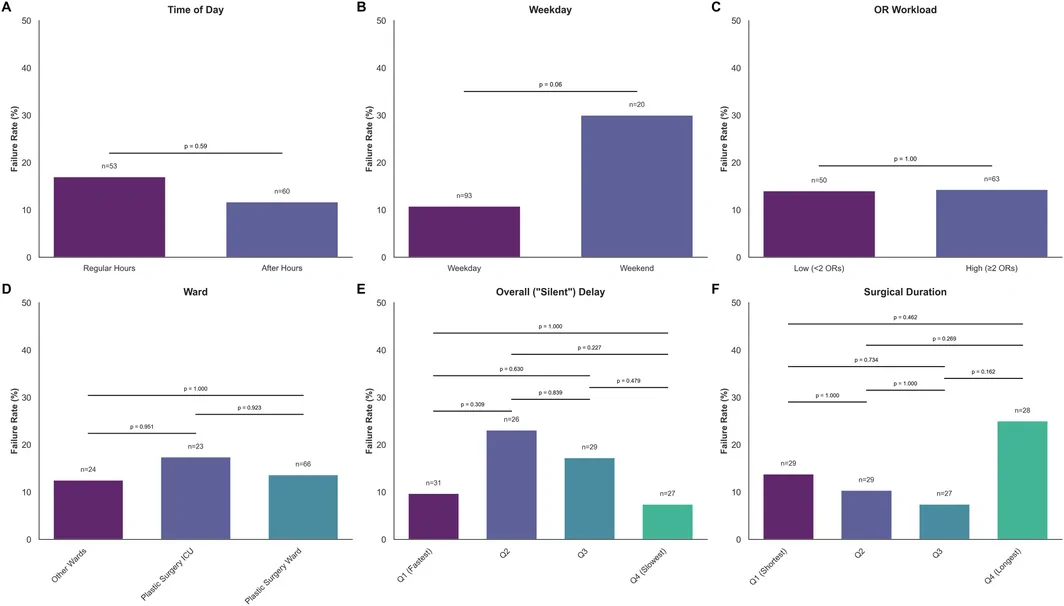
Failure rate (%) after free‐flap re‐exploration across workflow and timing factors. Bars depict the proportion of re‐explorations resulting in flap loss, with group sizes (*n*) shown on the bars (A) time of day (regular hours vs. after hours), (B) day of week (weekday vs. weekend, with a higher failure rate on weekends; *p* = 0.06), (C) operating room workload (low, < 2 concurrent ORs, vs. high, ≥ 2 concurrent ORs), (D) ward at the time of take‐back (other wards, Plastic Surgery ICU, Plastic Surgery Ward), (E) overall (“silent”) delay quartiles defined by registration‐to‐incision time (Q1 fastest to Q4 slowest) and (F) surgical duration quartiles defined by incision‐to‐closure time (Q1 fastest to Q4 slowest). All times are in minutes.

When surgical delay, defined as the time from decision to incision, was analyzed by quartiles, an unexpected pattern emerged (Figure 5): Failures peaked in the middle quartiles (Q2–Q3) and were lowest with the longest delays (Q4 7.4%). Failure proportions followed an inverted U‐shape with the lowest at the extremes and higher in the middle (Q1 9.7%, Q2 23.1%, Q3 17.2%, Q4 7.4%). However, the mentioned trend demonstrated no significance (*p* = 0.33; all pairwise *p* > 0.10, lowest for Q1 vs. Q4; *p* = 0.10).

To ensure that no relevant associations were overlooked, we additionally examined global correlations between all process and system variables using a Pearson‐based correlation heatmap (Figure S1). This analysis did not identify additional robust or clinically meaningful correlations beyond those described above and is therefore intended as a descriptive, hypothesis‐generating overview to guide future analyses.

## 4 Discussion

In this single‐center cohort, pre‐incision logistics were not the elements that separated salvaged from failed cases. Instead, differences clustered around clinical and technical complexity with longer anesthesia lead time and longer operative duration in the failure group. Global registration‐to‐incision times were identical (≈78 min vs. 77 min) representing a highly structured process with minimal deviation in itself. This consistency is a good quality indicator, but it also limits the ability to identify which parts of the surgical delay promote flap failure.

The case mix of this cohort warrants contextual consideration. Most reconstructions involved the extremities rather than the breast, and extremity free flaps are well recognized to carry higher complication and re‐exploration rates, attributable to more complex recipient vessel anatomy, trauma‐related endothelial injury, and greater thrombogenic burden (Vemula et al. 2017; Odorico et al. 2023). Notably, the proportion of re‐explorations occurring beyond 72 h (15%) exceeded the 11.6% reported by Bigdeli et al. in a comparable lower‐extremity cohort (Bigdeli et al. 2019), despite our more restrictive inclusion criteria, which excluded hematoma‐related re‐explorations unless hematoma directly precipitated vascular compromise such as venous compression. This difference may reflect variations in patient characteristics, underlying pathology, or overall case complexity. Two further institutional factors likely explain the comparatively high re‐exploration and salvage rate. Around‐the‐clock hourly flap monitoring supports the high salvage rate by enabling early recognition of compromise, while an explicit low‐threshold return‐to‐OR policy additionally raises the number of re‐explorations and enables intervention before irreversible damage occurs.

The time from registration‐to‐incision is best appreciated when our findings are situated within a broader Recognition‐to‐Reperfusion framework, which conceptualizes free‐flap salvage as a continuum of six phases with the recognition of vascular compromise, pathway activation, transport to the operating suite, anesthesia optimization, incision, and reperfusion (Kamali et al. 2021; Hosein et al. 2016; Tenorio et al. 2009; Chen et al. 2007). In this scheme, the registration‐to‐incision represents only one part in the recognition‐to‐reperfusion framework.

The present data suggest that surgical delay alone may not be the primary determinant of salvage success. At our institution, variability in the pre‐incision process was minimal, with working times near practical minima. At first glance, this seems to conflict with Bui et al. (2007) who argued that prolonged delay is the major driver of flap failure and with Gupta et al. (2024), who reported a decline in salvage probability from 57% to 11% when return to theater exceeded 3 h after recognition. These studies support the dogma that “time is tissue,” which our findings do not dispute. Rather, in our cohort, total pre‐incision intervals were consistently well below the critical three‐hour threshold, likely explaining the absence of detectable time dependence and is itself a reflection of an optimized pathway.

This interpretation is further supported when considering the Recognition‐to‐Reperfusion continuum in its entirety. Ahmad et al. identified a 5 h window from the onset of the first clinical signs to reperfusion as a practical threshold distinguishing successful salvages from failures (Ahmad et al. 2015). Although we did not measure recognition‐to‐reperfusion directly, our cohort's median pre‐incision overall or “silent” delay was 77 min, which would represent ~26% of a 5‐h window. Within that interval, realistic time savings are modest because patient transport, anesthesia optimization, and essential setup already operate near practical minima. Incremental compression of these steps is therefore unlikely to yield meaningful outcome gains compared with the far larger potential benefit of earlier recognition and faster pathway activation. Notably, anesthesia lead time showed a large median gap, warranting studies to determine whether prolonged lead times causally contribute to flap failure or simply mark higher‐complexity cases or procedural challenges requiring extended physiologic optimization prior to incision.

Finally, the observed *U*‐shaped association between operative duration and failure rate may support two plausible mechanisms: Short procedures may reflect early recognition of inherently non‐salvageable pathology, whereas prolonged surgeries represent technically complex rescues associated with cumulative physiological stress. A similar pattern was observed for pre‐incision delay, suggesting that prolonged intervals may reflect diagnostic uncertainty or evolving compromise in borderline cases, whereas clearly compromised flaps typically proceed to immediate exploration rather than indicating system inefficiency.

The timing of re‐exploration added a further layer to this picture. Although night‐time surgery was not associated with higher risk in our cohort and may even have been favorable, the observed weekend trend toward higher failure proportions aligns with broader literature describing performance and outcome differences during off‐hours (Naumann et al. 2024; Yang et al. 2019; de Cordova et al. 2012). While a substantial body of evidence shows that clinician fatigue, circadian disruption, and sleep deprivation impair psychomotor performance, executive function, and vigilance (Aylin et al. 2013), we did not observe an after‐hours effect and could not confirm Lee et al.'s report of higher flap loss for surgeries performed between 16:00 and 7:00 (Lee and Mun 2013).

Beyond microsurgery, numerous studies have linked after‐hours surgery to higher complication and mortality rates, even after adjustment for case urgency and complexity (Cortegiani et al. 2020; Halvachizadeh et al. 2019; Rahim et al. 2025), with reduced staffing levels and limited consultant availability proposed as potential contributing factors (Tucker et al. 2025; Arabadzhiyska et al. 2013). In our cohort, the elevated weekend failure proportion is unlikely to reflect such a staffing‐ or response‐related deficit: pre‐incision logistics on weekends were comparable to weekdays, and our protocol maintains uniform hourly physician‐led monitoring throughout the entire postoperative period, including overnight and weekend hours (Table S2). A more plausible explanation lies in the postoperative timing of the ischemic event itself. Because most primary free‐flap reconstructions were performed during weekdays, weekend salvage procedures inevitably occurred later in the postoperative course; consistent with this, all four Sunday cases presented ≥ 48 h after index surgery, a time interval associated with reduced salvage success (Shen et al. 2021). Nonetheless, the observed trend aligns with a well‐described “weekend effect” in the surgical literature and underscores that the most modifiable risk factor across all temporal contexts is not the clock, but the consistency of experienced decision‐making at the bedside (Hallas and Ellingsen 2006).

Our findings also indicate the possibility that the decisive delay may occur before clinical recognition rather than within the hospital workflow that follows. The discrepancy between nearly identical registration‐to‐incision timelines and divergent outcomes supports the notion that subclinical vascular compromise may progress for a considerable period before triggering overt signs. This pre‐detection window is rarely quantified in existing literature and may represent the true target for future monitoring innovation. Current monitoring methods, such as handheld acoustic Doppler, are widely used but assess inflow rather than the flap's own anastomotic vessels (Khatri et al. 2017; Salgado et al. 2009). For this reason, adjuncts such as color duplex ultrasonography (Chao and Lamp 2014), implantable Doppler flow couplers (Troob et al. 2021), and emerging spectral or hyperspectral imaging modalities are increasingly explored to reduce time until detection of compromise (Abdou et al. 2020; Yuen and Feng 2000). The effectiveness of these technologies has still to be evaluated in larger clinical studies, but so far underscores the need to shorten the biologically harmful interval preceding formal recognition rather than focusing solely on in‐hospital logistics (Knoedler et al. 2023).

This study has several limitations. First, its retrospective, single‐center design limits external validity and introduces potential selection and information bias. Time‐stamp accuracy relied on documentation in the EPR, which may not perfectly capture parallel processes such as pre‐alerting of teams or manual preparation activities. Although implausible intervals were cross‐checked against operating‐room logs, small timing inaccuracies cannot be excluded. The sample size was small, limiting statistical power to detect subtle effects. Moreover, unmeasured confounders like patient comorbidities and micro‐surgical expertise may have influenced both operative duration and outcome. However, the aim of this research was to assess the effect of process‐related delays rather than patient‐driven factors. Finally, our institution's standardized salvage pathway compressed variability across process times, which may explain the absence of statistically significant differences between salvage and failure groups. Accordingly, our findings should be interpreted cautiously within the context of a system with few extended delays, rather than as contradictory to studies examining much longer pre‐incision intervals. Consequently, the findings should be interpreted as descriptive benchmarks rather than definitive causal inferences. Moreover, limited sample size, particularly in subgroup analyses, restricts the ability to detect small effects related to workload intensity or staffing variability.

## 5 Conclusions

In this cohort, pre‐incision logistical intervals such as patient transport and operating‐room preparation were not associated with free‐flap salvage failure. Instead, differences between successful and failed cases after recognition of flap compromise appeared to reflect case complexity rather than modifiable in‐hospital delays. These findings suggest that efforts to improve outcomes should prioritize earlier detection of vascular compromise and rapid activation of salvage pathways, rather than further compressing already optimized operative logistics.

## Funding

The authors have nothing to report.

## Ethics Statement

This study was conducted in accordance with the Declaration of Helsinki. As anonymous data have been used, no ethical approval was needed.

## Conflicts of Interest

The authors declare no conflicts of interest.

## Supporting information


**Figure S1:** Pearson correlation matrix of process, system, and outcome variables. Heatmap of pairwise Pearson correlations in the re‐exploration cohort (*n* = 113). Continuous variables represent process intervals and surgical duration. Categorical variables were one‐hot encoded. Correlations involving categorical variables correspond to point‐biserial or phi coefficients. Strong correlations between overlapping time intervals reflect their shared timeline components. No process or system variable showed a meaningful association with outcome. Complementary one‐hot categories may show structural *r* = −1. The matrix is descriptive. No significance testing or multiple‐comparison correction was performed.

**Table S1:** Outcomes of free‐flap re‐exploration stratified by flap class. Percentages are within each row. Fisher's exact test (two‐sided): any loss (partial or total) OR = 0.68, *p* = 0.776; total loss only *p* = 1.00.

**Table S2:** Comparison of weekday and weekend re‐exploration cases regarding postoperative timing, pre‐incision logistics, and off‐hours frequency. Weekday = Monday through Friday; weekend = Saturday and Sunday. POD = postoperative day, defined as the interval in days from index free‐flap surgery to re‐exploration. Registration‐to‐incision time was computed from emergency registration to start of the re‐exploration procedure. Off‐hours = re‐exploration commenced between 20:00 and 07:00. Mann–Whitney *U* tests (two‐sided) were used for continuous variables given right‐skewed distributions; Fisher's exact test was used for categorical comparisons. Median POD did not differ in central tendency but the underlying distribution differed significantly (*p* = 0.001), with weekend cases skewed toward later postoperative days. Pre‐incision logistics did not differ between weekday and weekend cases (*p* = 0.310), and off‐hours frequency was comparable (*p* = 0.179), indicating that the elevated weekend failure proportion was not attributable to delayed in‐hospital response or to a higher proportion of overnight procedures.

## Data Availability

Data supporting the findings of this study are available from the corresponding author upon request.
