# Supplementary material for: Pre‐Incision Time Intervals and Outcomes of Free Flap re‐Exploration: A Retrospective Single‐Center Study
Source: Microsurgery. 2026 Sep 11;46(6):e70292. doi: 10.1002/micr.70292 (PMC13563678; doi:10.1002/micr.70292)
Supplement: Supplementary file 1 [file MICR-46-e70292-s001.docx]

| **Metric** | **Weekday** | **Weekend** | **Test, p-value** |
| --- | --- | --- | --- |
| **Total cases, n** | 93 | 20 |  |
| **Median POD at revision, d** | 1.0 | 1.0 | Mann-Whitney U,  p = 0.001 |
| **Median registration-to-incision, min** | 77 | 68 | Mann-Whitney U,  p = 0.310 |
| **Off-hours cases (20:00–07:00), n** | 29 | 3 | Fisher exact,  p = 0.179 |

***Supplementary Table S2.*** *Comparison of weekday and weekend re-exploration cases regarding postoperative timing, pre-incision logistics, and off-hours frequency. Weekday = Monday through Friday; weekend = Saturday and Sunday. POD = postoperative day, defined as the interval in days from index free-flap surgery to re-exploration. Registration-to-incision time was computed from emergency registration to start of the re-exploration procedure. Off-hours = re-exploration commenced between 20:00 and 07:00. Mann-Whitney U tests (two-sided) were used for continuous variables given right-skewed distributions; Fisher's exact test was used for categorical comparisons. Median POD did not differ in central tendency but the underlying distribution differed significantly (p = 0.001), with weekend cases skewed toward later postoperative days. Pre-incision logistics did not differ between weekday and weekend cases (p = 0.310), and off-hours frequency was comparable (p = 0.179), indicating that the elevated weekend failure proportion was not attributable to delayed in-hospital response or to a higher proportion of overnight procedures.*
