# Supplementary material for: Pre‐Incision Time Intervals and Outcomes of Free Flap re‐Exploration: A Retrospective Single‐Center Study
Source: Microsurgery. 2026 Sep 11;46(6):e70292. doi: 10.1002/micr.70292 (PMC13563678; doi:10.1002/micr.70292)
Supplement: Supplementary file 2 [file MICR-46-e70292-s003.docx]

| **Flap class** | **Salvage,  n (%)** | **Partial loss,  n (%)** | **Total loss,  n (%)** | **Any loss,  n (%)** |
| --- | --- | --- | --- | --- |
| Muscle flap  (n = 32) | 28 (87.5%) | 0 (0) | 4  (12.5) | 4  (12.5) |
| Perforator flap  (n = 81) | 67 (82.7) | 2 (2.5) | 12  (14.8) | 14  (17.3) |
| Total  (n = 113) | 95 (84.1) | 2 (1.8) | 16  (14.2) | 18  (15.9) |

***Supplementary Table S1:*** *Outcomes of free-flap re-exploration stratified by flap class. Percentages are within each row. Fisher's exact test (two-sided): any loss (partial or total) OR = 0.68, p = 0.776; total loss only p = 1.00.*
